# Deciphering glutamine metabolism patterns for malignancy and tumor microenvironment in clear cell renal cell carcinoma

**DOI:** 10.1007/s10238-024-01390-4

**Published:** 2024-07-06

**Authors:** Gengrun Wu, Teng Li, Yuanbiao Chen, Shiqi Ye, Siqi Zhou, Xi Tian, Aihetaimujiang Anwaier, Shuxuan Zhu, Wenhao Xu, Xiaohang Hao, Dingwei Ye, Hailiang Zhang

**Affiliations:** 1 Department of Urology, Fudan University Shanghai Cancer Center, Shanghai, 200032 People’s Republic of China; 2 Department of Oncology, Shanghai Medical College, Fudan University, Shanghai, 200032 People’s Republic of China; 3 Shanghai Genitourinary Cancer Institute, Shanghai, 200032 People’s Republic of China; 4 Department of Urology, The Affiliated Taian City Central Hospital of Qingdao University, Taian, 271000 People’s Republic of China; 5 Affiliated Hospital of Youjiang Medical University for Nationalities, Baise, 533000 People’s Republic of China

**Keywords:** Clear cell renal cell carcinoma, Glutamine metabolism, Tumor microenvironment, Prognosis, Immunotherapy response, ALDH18A1

## Abstract

Clear cell renal cell carcinoma (ccRCC) is the most common subtype of kidney cancer characterized by metabolic reprogramming. Glutamine metabolism is pivotal in metabolic reprogramming, contributing to the significant heterogeneity observed in ccRCC. Consequently, developing prognostic markers associated with glutamine metabolism could enhance personalized treatment strategies for ccRCC patients. This study obtained RNA sequencing and clinical data from 763 ccRCC cases sourced from multiple databases. Consensus clustering of 74 glutamine metabolism related genes (GMRGs)- profiles stratified the patients into three clusters, each of which exhibited distinct prognosis, tumor microenvironment, and biological characteristics. Then, six genes (SMTNL2, MIOX, TMEM27, SLC16A12, HRH2, and SAA1) were identified by machine-learning algorithms to develop a predictive signature related to glutamine metabolism, termed as GMRScore. The GMRScore showed significant differences in clinical prognosis, expression profile of immune checkpoints, abundance of immune cells, and immunotherapy response of ccRCC patients. Besides, the nomogram incorporating the GMRScore and clinical features showed strong predictive performance in prognosis of ccRCC patients. ALDH18A1, one of the GRMGs, exhibited elevated expression level in ccRCC and was related to markedly poorer prognosis in the integrated cohort, validated by proteomic profiling of 232 ccRCC samples from Fudan University Shanghai Cancer Center (FUSCC). Conducting western blotting, CCK-8, transwell, and flow cytometry assays, we found the knockdown of ALDH18A1 in ccRCC significantly promoted apoptosis and inhibited proliferation, invasion, and epithelial-mesenchymal transition (EMT) in two human ccRCC cell lines (786-O and 769-P). In conclusion, we developed a glutamine metabolism-related prognostic signature in ccRCC, which is tightly linked to the tumor immune microenvironment and immunotherapy response, potentially facilitating precision therapy for ccRCC patients. Additionally, this study revealed the key role of ALDH18A1 in promoting ccRCC progression for the first time.

**Supplementary Information:**

The online version contains supplementary material available at 10.1007/s10238-024-01390-4.

## Introduction

In 2022, China reported approximately 73,700 new kidney cancer cases [1]. Renal cell carcinoma (RCC) ranked eighth in the number of new tumor cases in the United States in 2024 [2]. Clear cell renal cell carcinoma (ccRCC) constitutes about 75% of all RCC [3, 4]. The treatment landscape of ccRCC has undergone a tremendous transformation in recent years, thanks to the advent of immunotherapy and the combination of immune checkpoint inhibitors (ICIs) with tyrosine kinase inhibitors (TKIs) [5]. These regimens include nivolumab with ipilimumab or cabozantinib, pembrolizumab with axitinib or lenvatinib, and avelumab with axitinib [5]. Toripalimab combined with axitinib resulted in a significantly longer overall response rate (ORR) and progression-free survival (PFS) than axitinib alone as first-line therapy in advanced untreated ccRCC in a phase III clinical trial conducted in China [6]. Moreover, a latest analysis of 175 RCC patients revealed that IO-TKI therapy improved PFS compared to IO-IO therapy [7]. However, evidence from post hoc biomarker analysis of clinical trials and routine clinical practice demonstrates heterogeneous response to these treatment strategies, underscoring the necessity to pinpoint characteristics for patients stratification [8]. Currently, the potential prognostic value of single genetic mutation such as *PBRM1* has been explored in various therapeutic settings, especially to predict response to immune checkpoint blockade (ICB) [9]. Meanwhile, several gene expression signatures depicting the complex interplay in TME, as determined by tumor molecular profiling, have been suggested to direct the precise clinical treatment for patients with ccRCC [10–14]. A subsequent investigation to the IMmotion150 trial has explored the genetic characteristics of patients with ccRCC, and three unique gene signatures (Angiogenesis, T-effector, and Myeloid inflammation) were found to be closely correlated with therapeutic response in different treatment arms [11]. Xu et al. [14] investigated the distinctive prognostic mutational landscape of Chinese ccRCC patients and suggested a prognostic signature composed of seven genes that indicated a poor prognosis and was associated with responsiveness to ICB. These results highlighted the significance of utilizing multi-gene expression signature that captures TME characteristics as a guiding tool for precision therapy in ccRCC.

To fulfill their own proliferative demands, certain solid tumor cells alter their metabolic patterns and rewire nutrient uptake and metabolic pathways to supply the bioenergetic and redox needs for cancer cell biosynthesis. This process, known as metabolic reprogramming, is driven by the inactivation of tumor suppressor genes and the activation of oncogenes [15]. The Warburg Effect, also recognized as aerobic glycolysis, is the most prominent example of metabolic reprogramming in cancer. It describes the glucose metabolism of tumor cells: under normal oxygen conditions, these cells continue to rely on glycolysis for energy production. This means that, regardless of oxygen availability, pyruvate is predominantly converted to lactate to generate ATP. This shift of tumor cells from oxidative phosphorylation to glycolysis in glucose utilization is considered a hallmark of tumor. It is widely accepted that this metabolic adaptation confers growth advantages to tumor cells, aids in evading apoptosis, and contributes to the progression of cancer in vivo [16]. Moreover, glycolysis induces cancer-associated fibroblasts to secrete lactate and hydrogen ions, fostering an immune-suppressive microenvironment [17]. RCC, a prevalent tumor within the urinary system, serves as a prime example of metabolic reprogramming. This malignancy is characterized by alterations and disruptions in lots of metabolic pathways, such as glycolysis, the tricarboxylic acid (TCA) cycle, glutamine and tryptophan metabolism. As a result, RCC is also recognized as a metabolic disease [18]. To ccRCC, metabolic reprogramming is also a hallmark, and alterations in gene expression patterns result in substantial metabolic alterations that fuel tumor progression [19]. Reports indicate that as many as 90% of ccRCC cases exhibit inactivation mutations in the von Hippel-Lindau (VHL) [20]. VHL protein is an enzyme that functions as an E3 ubiquitin ligase, which targets hydroxylated HIF-1/HIF-2 α for ubiquitination and subsequent degradation under normal oxygen levels [21]. VHL mutations can result in the accumulation of HIF-1/HIF-2 α, leading to the dysregulation of the HIF signaling pathway [22]. This abnormal activity promotes the initiation and progression of ccRCC by multiple pathways including reprogramming the metabolism of tumor cells [23]. Glutamine addiction is one of the main changes in ccRCC cellular metabolism [24].

Glutamine is the most abundant non-essential amino acid in human blood, serving as a source of carbon and nitrogen for cellular biosynthesis and energy for cellular proliferation. Glutamine enters tumor cells via specific transporters such as SLC1A5 [25], and is then catalyzed to produce glutamate through glutaminase (GLS) [26]. Glutamate is then further metabolized by glutamine dehydrogenase (GDH) to produce α-ketoglutarate (α-KG). The α-KG enter TCA cycle, contributing to ATP production to provide energy to cells through the oxidative phosphorylation pathway [27]. Consequently, glutamine serves as a carbon source to fuel the TCA cycle, which is a central metabolic pathway within cells, primarily occurring in the mitochondrial matrix. Additionally, glutamine’s complete oxidation to generate ATP allows it to be used as a nitrogen source for synthesizing nucleotides and various non-essential amino acids [28]. Furthermore, glutamine is crucial for preserving cellular redox equilibrium, as its metabolite glutamate contributes to the synthesis of glutathione, a vital cellular antioxidant [29]. In contrast to normal cells, tumor cells cannot meet their rapid proliferation demands through self-synthesis of glutamine [24].

The metabolism of glutamine can impact tumor cells directly, consequently influencing antitumor immune response. For instance, when the glutamine was deprived, expression of PD-L1 on tumor cell surfaces elevated through activation of the EGFR/ERK/C-Jun pathway, thereby inhibiting immune response against tumors in bladder and kidney tumor cells [30, 31]. Meanwhile, the tumor immune microenvironment (TME) can also be modulated by glutamine metabolism. In the case of glutamine-dependent ccRCC, tumor cells’ intense uptake of glutamine leads to a scarcity of extracellular glutamine in the local environment. This depletion triggers the activation of hypoxia-inducible factor 1α and prompts IL-23 secretion by tumor-associated macrophages. The secretion of IL-23 subsequently stimulates regulatory T cells to proliferate and suppress cytotoxic lymphocytes, which in turn inhibits antitumor capabilities [32]. However, utilizing multi-gene signatures to dissect the influence of glutamine metabolism related genes (GMRGs) in TME and prognosis of patients with ccRCC still lacks clarification.

In the current study, integrated analysis of the impact of GMRGs in ccRCC was conducted. Furthermore, we had established a glutamine metabolism related signature that exhibits a significant correlation with patient prognostic outcomes, features of the tumor microenvironment (TME), and responses to immunotherapy in individuals afflicted with ccRCC. Our findings provided a deeper understanding that could guide future research about glutamine metabolism in ccRCC, which may contribute to precision therapy for ccRCC patients. As far as we are aware, our research marks the initial study to clarify the tumor-promoting function of ALDH18A1 (P5CS) in ccRCC, indicating its potential as a therapeutic target for this disease.

## Materials and methods

### Sample collection and data acquisition

We utilized the publicly available information of ccRCC patients from The Cancer Genome Atlas database, Clinical Proteomic Tumor Analysis Consortium database, International Cancer Genome Consortium database, and European Molecular Biology Laboratory database, including TCGA-KIRC, CPTAC-3, RECA-EU, and E-MTAB-3267 cohorts. We selected the same genes from four different cohorts, and performed a log2 (TPM + 1) transformation on them. The “sva” R package was used to integrate all samples [33]. Additionally, we procured datasets pertaining to copy number variations (CNVs) and somatic mutations from the TCGA database. The comprehensive baseline clinical details are aggregated and presented in Table S1**.**

### Capture of GMRGs and cluster analysis

We acquired the GMRGs set from the Molecular Signatures Database. The ‘GOBP_GLUTAMINE_FAMILY_AMINO_ACID_METABOLIC_PROCESS’ gene set is taken to intersect with our data obtained previously (Table S2). We utilized the “ConsensusClusterPlus” package to perform consensus clustering, thereby patients were subtyped [34].

### Exploration of TME in different metabolism patterns

We applied CIBERSORT algorithm to determine the relative abundances of immune cell types within TME [35]. We examined and contrasted the expression levels of immune checkpoint (ICPs) across selected subgroups. Besides, we utilized the ESTIMATE algorithm to determine the stromal and immune cell content [36].

### Identification of differential expression genes (DEGs) and functional enrichment analysis

To identify DEGs in the distinct GMRClusters, we used the “limma” package, and the criteria was set at FDR < 0.001 and |log2-fold change (FC)|> 0.8 [37]. Gene Ontology (GO) and Kyoto Encyclopedia of Genes and Genomes (KEGG) enrichment analysis were conducted with the “clusterProfiler” package, premised on the p-value and FDR thresholds of < 0.05 [38]. We performed the gene set variation analysis (GSVA) among distinct clusters with the KEGG gene set (c2.cp.kegg.v2023.1.Hs.symbols), and the criteria was set at |log2-fold change (FC)|> 0.1 and FDR < 0.05 [39].

### Development of a glutamine metabolism related predictive signature

The DEGs underwent univariate Cox (uniCox) regression to assess the prognostic significance for overall survival, deeming a P value of less than 0.05 as significant. DEGs with a defined *P* value (*P* < 0.001) were subsequently subjected to LASSO and multivariate Cox analyses construct the prognostic signature (GMRScore). The GMRScore was computed with the RiskGenes (SMTNL2, MIOX, TMEM27, SLC16A12, HRH2, and SAA1) as described: GMRScore = ∑expression of RiskGene* corresponding coefficient. The ccRCC patients were categorized into two GMRGroups, using the median GMRScore as the threshold.

### Verification of a GMRScore

We conducted a stratified survival analysis on patients categorized into distinct subgroups to evaluate the reliability of the GMRScore. The Tumor Immune Dysfunction and Exclusion (TIDE) score data were obtained from the TIDE database to forecast the response to ICB, with the low-scoring groups being considered highly responsive [40]. Besides, two cohort of patients treated with anti-PD1 ICB were used to validate the response to ICB of the GMRScore. In the cohort reported by David Liu, 121 metastatic melanoma patients received nivolumab or pembrolizumab [41]. The David A. Braun cohort consisted of 181 advanced clear cell renal cell carcinoma (accRCC) patients received nivolumab [42].

### Establishment and validation of a nomogram

We eatablished a nomogram integrating GMRScore with clinical features to forecast the OS probabilities for patients with ccRCC utilizing a suite of advanced R programming language packages. These include the “rms” package, which is instrumental in regression modeling, the “regplot” package for its advanced plotting capabilities, and the “survival” package, which is essential for conducting survival analysis.

### Cell lines and reagents

The 786-O and 769-P, two human ccRCC cell lines, were obtained from Shanghai Institute of Life Sciences. They were incubated in RPMI-1640 medium (Gibco) added 10% FBS (Gibco). Cells were grown in a 5% CO_2_ environment at 37 °C.

### Cell counting kit (CCK)-8 assay

The cells were plated in a density of 5000 cells per well. Cells were cultured for 5 days in total, and their viability was assessed using the CCK-8 everyday, following the protocol (Beyotime). The optical density (OD) was measured at a wavelength of 450 nm.

### Cell transfection

When the cells reached 80% density, transfection could commence. The negative control and P5CS siRNA were blended with Lipofectamine® 2000 reagent (Thermo Fisher Scientific) following manufacturer’s instructions.

### Western blotting assay

Cells were gently scraped into a RIPA buffer (sourced from Beyotime, Nanjing, China) that was supplemented with protease inhibitors (supplied by KeyGEN, Nanjing, China). The primary antibodies used includes P5CS (17719–1-AP,1:1000, Proteintech), N-cadherin (22018–1-AP, 1:5000, Proteintech), SNAI1 (13099–1-AP, 1:1000, Proteintech), Vimentin (10366–1-AP, 1:5000, Proteintech), and GAPDH (60004–1-Ig, 1:50000, Proteintech).

### Transwell assay

In summary, 5 × 10^3^ cells that were stably infected were resuspended with serum-free medium and planted into upper layer of a 24-well transwell filter with Matrigel. The bottom compartment received 800 μL of medium enriched with 20% FBS. After 24-h incubation, the chambers underwent three PBS washes, each lasting five minutes. Cells in the lower chambers were then stabilized with 4% paraformaldehyde for half an hour, while the non-invasive cells above were removed using cotton balls. Next, a 20-min staining process with 1% crystal violet was performed at room temperature. After another series of three 5-min PBS washes, the cells in the lower section were examined and photographed using an inverted microscope. The ImageJ software was utilized to tally the number of invaded cells in each viewed area.

### Cell apoptosis analysis using flow cytometry

After cells were transfected, the cells were trypsinized without EDTA. 1–10 × 10^5^ cells were resuspended in 500ul 1X binding buffer from the FITC Annexin V Apoptosis Detection kit (cat#:AP101-100-kit; Multi Sciences). Then 5 µl Annexin V-FITC and 10 µl PI staining solution were incorporated and the samples were cultured at room temperature for 5 min in the absence of light. The cells underwent flow cytometry analysis using a FACS Calibur flow cytometer.

### Statistical analysis

The entirety of the statistical computations and graphical representations were performed utilizing R version 4.3.2 and GraphPad Prism 8.0.2. *P* value < 0.05 was established to denote statistical significance, unless specified differently.

## Results

### GMRGs in ccRCC: multi-omics landscape

**Fig. 1:**
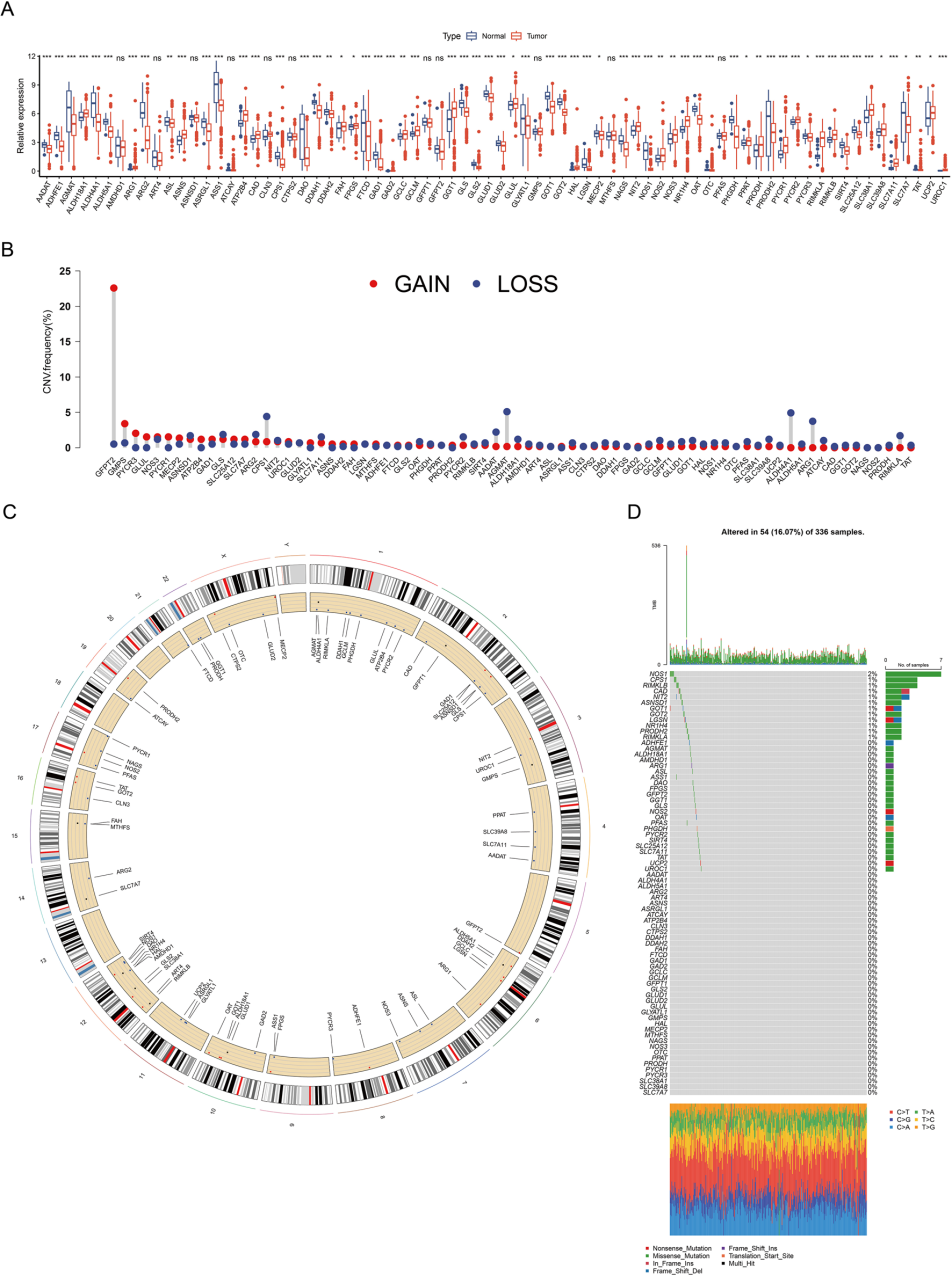
Multi-omics landscape of glutamine metabolism related genes (GMRGs) in Clear Cell Renal Cell Carcinoma (ccRCC). **A** Boxplot of the expressions of the 74 GMRGs in the TCGA-KIRC cohort. **B** Copy number variations (CNVs) of the GMRGs in ccRCC from the TCGA-KIRC cohort. **C** Location of CNV alteration of GMRGs on chromosomes in ccRCC from the TCGA-KIRC cohort. **D** Mutation frequency and types of GMRGs on chromosomes in ccRCC from the TCGA-KIRC cohort. ns, not significant, *p < 0.05, **p < 0.01, ***p < 0.001

We first utilized the TCGA-KIRC dataset to assess mRNA expressions of 74 GMRGs (Table S2) in tumor and normal specimens. Among them, 63 GMRGs exhibited differential expressions (Fig. 1A). This differential expression indicates that these genes could be involved in advancement of ccRCC. In addition to expression analysis, we conducted a thorough investigation into the genomic CNVs within the GMRGs in ccRCC patients. Our findings indicated that, with the exception of five genes (GFPT2, CPS1, AGMAT, ALDH4A1, and ARG1), the frequency of CNVs across the GMRGs was relatively low (Fig. 1B). The specific locations of these CNVs on the chromosomes were meticulously mapped and visualized, providing a detailed genomic landscape of the alterations observed in ccRCC (Fig. 1C). At the genomic level, In 336 cases of ccRCC, 16.07% exhibited somatic mutations in GMRGs, notably, NOS1, CASP1, and RIMKLB displaying the most frequent mutations, while mutations in some genes were less common or absent (Fig. 1D). These findings indicate highly heterogeneous expression and mutation patterns of GMRGs in ccRCC. To assess clinical implications of our findings, we examined the predictive significance of GMRGs in ccRCC patients using data from four independent databases (Table S3).

### Generation of glutamine metabolic patterns and detection of variations across subpopulations

**Fig. 2:**
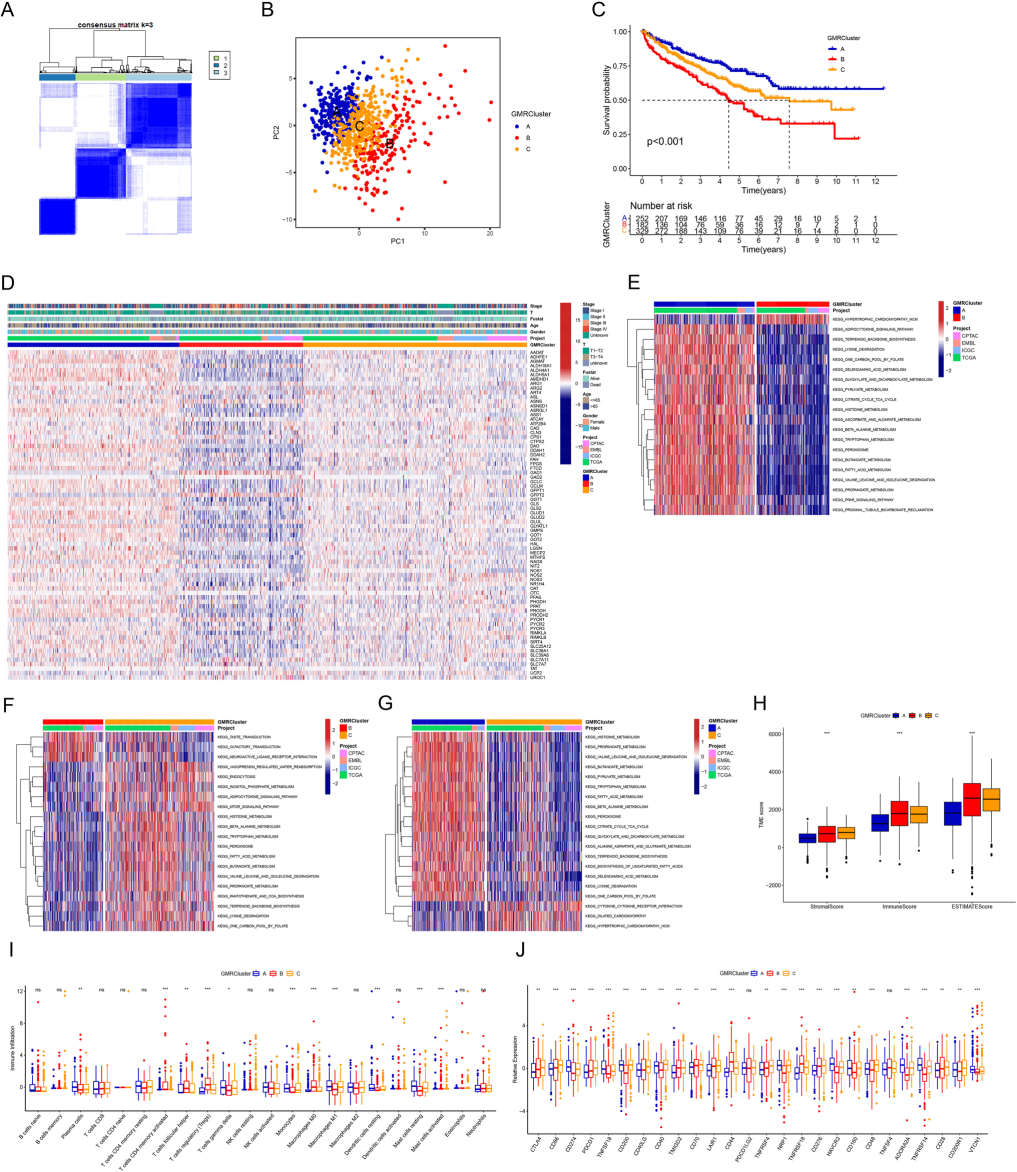
Generation and characteristics of glutamine metabolic patterns. **A** The unsupervised clustering diagram. **B** Principal component analysis (PCA) of GMRGs expression difference among three GMRClusters. **C** Survival analysis of three GMRClusters. **D** Heatmap of clinicopathological features and expressions of GMRGs among GMRClusters. **E–G** Gene set variation analysis (GSVA) of enriching biological pathways among three GMRClusters. **H** The differences in estimate, stromal, and immune scores of GMRClusters **I** The difference in infiltrating abundance of 22 immune cells among three GMRClusters. **J** The differences in expressions of ICPs among three GMRClusters. ns, not significant, *p < 0.05, **p < 0.01, ***p < 0.001

To delve deeper into understanding glutamine metabolic patterns in ccRCC, we employed we performed unsupervised consensus clustering for mRNA expression profiles of the 74 GMRGs and generated three groups of patients, forming GMRClusters. The optimal clustering parameter was determined to be 3, resulting in the distribution of patients into three distinct clusters: A (*n* = 252), B (*n* = 182), and C (*n* = 329), as depicted in Fig. 2A and detailed in Table S4. The picture had a minimal degree of consensus matrix crossover, reflecting high agreement and consistency. The CDF curves progressed smoothly, indicating a gradual and orderly accumulation of data without abrupt changes (Fig. S1). Principal Component Analysis (PCA) robustly confirmed the clear separation of GMRClusters, indicating distinct and well-defined distributions (Fig. 2B).

Survival analysis indicated that patients in cluster B experienced the shortest OS, whereas those in cluster A had the longest OS (Fig. 2C). Additionally, the gene expression patterns and clinicopathological variables of the three clusters were compared (Fig. 2D), revealing significant differences in GMRGs expression and clinical features. In opposition to cluster B, Cluster A exhibited the highest overall expression levels of GMRGs. GSVA analysis showed that among three clusters, cluster A exhibited heightened activity in amino acid metabolism pathways (lysine degradation, valine leucine and isoleucine degradation, glyoxylate and dicarboxylic acid metabolism, pyruvate metabolism, citric acid cycle and TCA cycle, tryptophan and histidine metabolism) while these pathways were downregulated in cluster B (Fig. 2E, F, and G).

To assess TME abundance, the ESTIMATE algorithm was employed, indicating fewer immune and stromal cell components in GMRCluster A (Fig. 2H). For immune cell infiltration, different GMRClusters showed significant enrichment differences (Fig. 2I). The expression levels of several key immune checkpoints (ICPs), such as PD-1 (PDCD1), PD-L1 (CD274), and CTLA-4, were found to exhibit significant differences. Specifically, within the GMRCluster A, the expression of PD-1 and CTLA-4 notably decreased (Fig. 2J). In summary, three glutamine metabolic patterns were identified, and their unique clinical features and TME underscored the importance of glutamine metabolism in shaping the clinical course and therapeutic responses of patients with ccRCC.

### Comprehensive analysis of clusters based on differentially expressed genes

**Fig. 3:**
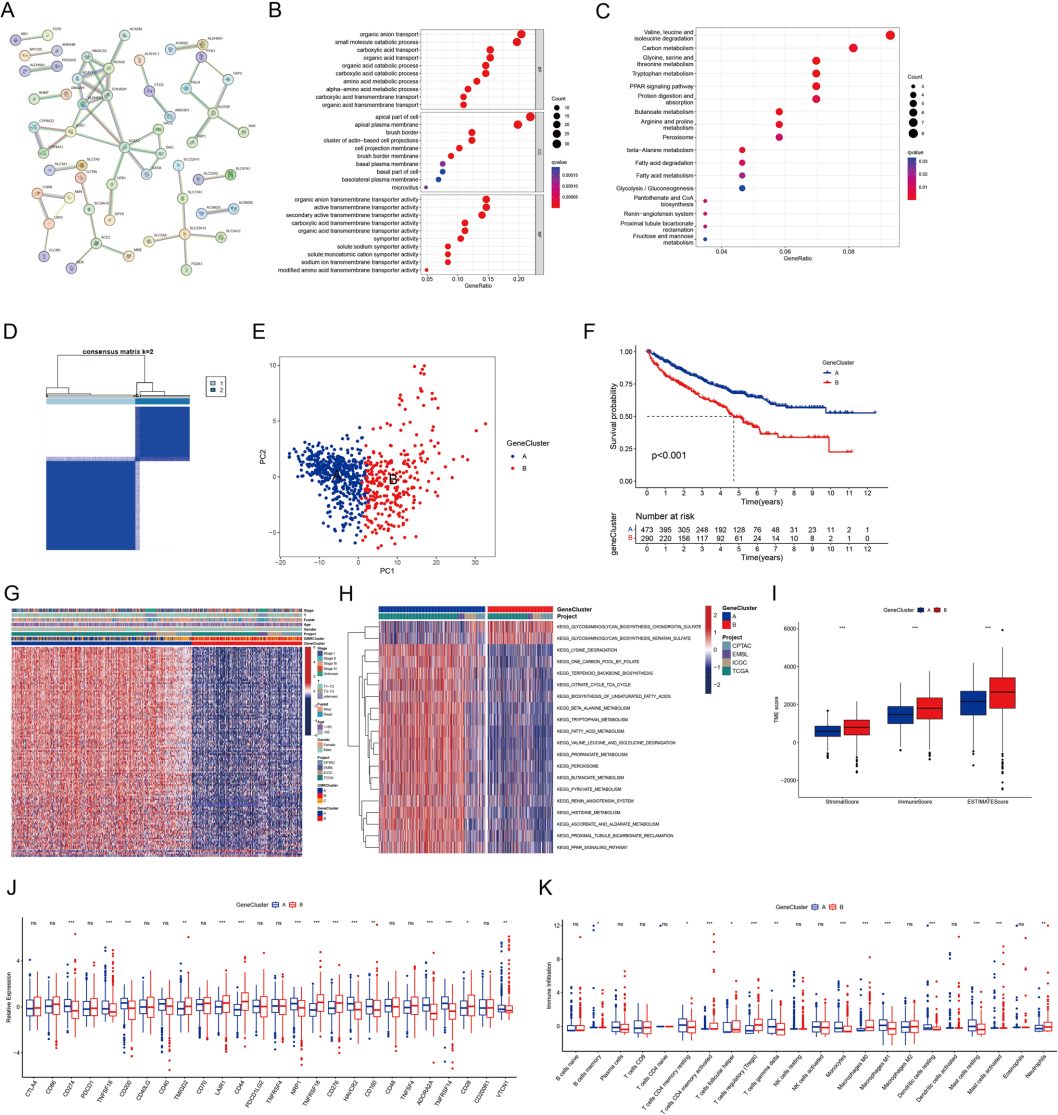
In-depth analysis of clusters derived from differentially expressed genes. **A** The protein protein interaction network of the DEGs from STRING database. **B** GO enrichment analysis of DEGs between two GeneClusters. **C** KEGG pathway analysis of DEGs between two GeneClusters. **D** Unsupervised clustering analysis of 130 prognosis-related genes. **E** PCA analysis of expression difference in 130 prognosis-related genes between two GeneClusters. **F** Survival analysis of two GeneClusters. **G** Heatmap of clinicopathological features and expression of prognosis-related genes between two GeneClusters. **H** GSVA enrichment analysis highlighting the biological pathway enrichment in two GeneClusters. **I** The differences in estimate, stromal, and immune scores of GeneClusters. **J** The differences in expression of ICPs between two GeneClusters. **K** The difference in infiltrating abundance of immune cells between two GeneClusters. ns—not significant, *p < 0.05, **p < 0.01, ***p < 0.001

To better grasp the molecular mechanism that shape glutamine metabolic patterns in ccRCC, we pinpointed 150 DEGs linked to GMRClusters (Table S5). The STRING database was employed to produce a PPI network within these DEGs, emphasizing the highest confidence and concealed disconnected nodes within the network (Fig. 3A). Next, GO analyses were conducted, providing substantial understanding of biological processes, molecular functions, and cellular components (Fig. 3B and Table S6). Concurrently, KEGG pathway analysis illustrated that the DEGs were enriched in pathways in particular valine, leucine, and isoleucine degradation, carbon metabolism, and Glycine, serine, and threonine metabolism (Fig. 3C and Table S6). Following this, 130 prognostically relevant genes were selected through uniCox (Table S7). The consensus clustering was subsequently utilized to partition patients into two distinct GeneClusters, utilizing the 130 prognostic genes (Fig. 3D, Fig. S2, and Table S7). These two GeneClusters were found to possess markedly different transcriptomic profiles, as evidenced by principal component analysis (PCA) (Fig. 3E). Further survival analysis indicated patients in GeneCluster A experienced a significantly longer overall survival period (Fig. 3F).

A heatmap displayed the disparities in clinical characteristics between the two GeneClusters, wherein most of the DEGs in GeneCluster A demonstrated relatively high expression levels (Fig. 3G). GeneCluster A was yielded to be significantly enriched in amino acid metabolism activation through GSVA analysis, including pathways such as lysine degradation, valine leucine, and isoleucine degradation, pyruvate metabolism, citric acid cycle and TCA cycle, tryptophan, and histidine metabolism (Fig. 3H and Table S8). The TME scores within the GeneClusters suggested lower fractions of immune cells and stromal cells as well as higher tumor purity in GeneCluster A (Fig. 3I). Subsequently, the expression profiles of immune checkpoint markers across various GeneClusters were analyzed (Fig. 3J). Notably, GeneCluster A exhibited a low presence of Tregs and neutrophils, along with a high infiltration level of macrophages M1, implying Immune activated tumor microenvironment and consistent with the observed favorable prognosis (Fig. 3K).

### Construction of a prognostic GMRScore

**Fig. 4:**
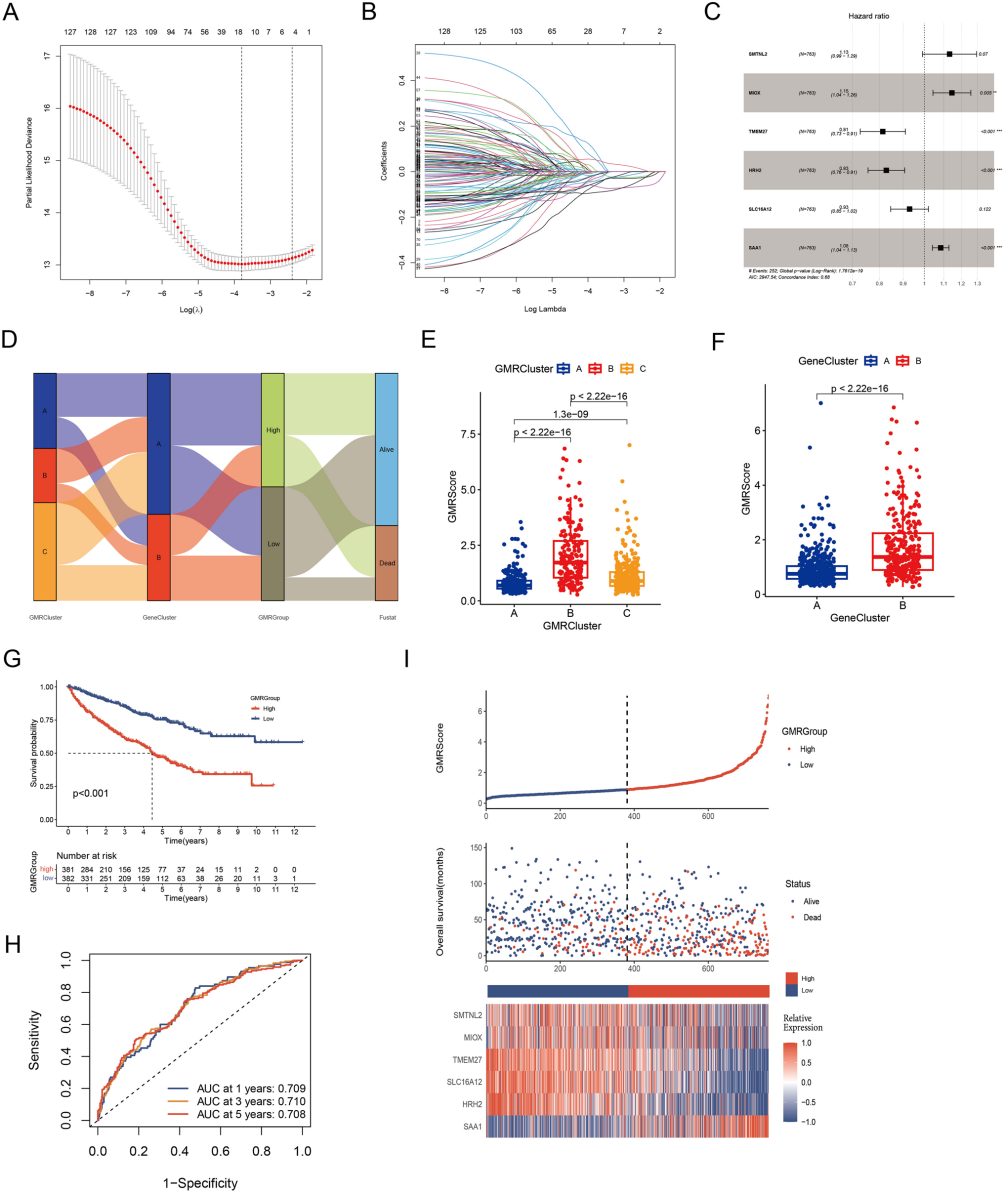
Construction of a glutamine metabolism related predictive signature (GMRScore). **A**, **B** Coefficient profiles and parameter selection in LASSO analysis. **C** Six prognostic genes were selected by the univariate Cox regression analysis. **D** Alluvial diagram of patients’ distributions in GMRClusters, GeneClusters, and GMRGroups. **E–F** Differences in GMRScore of GMRClusters and geneClusters. **G** Kaplan–Meier curves for the two GMRGroups. **H** ROC curves of GMRScore in predicting the 1-, 3-, and 5-year OS. **I** Distribution plots of the risk score, OS status, and heatmap of six gene expressions

To construct a glutamine metabolism related signature for prognosis indication of patients with ccRCC, we devised a scoring system, denoted as GMRScore. Employing LASSO and multivariate Cox analyses, we crafted an optimal predictive model based on 130 prognostic genes (Fig. 4A–C). We pinpointed six genes, encompassing SMTNL2, SAA1, MIOX, TMEM27, HRH2, and SLC16A12 (Table S9). Interestingly, SAA1 and MIOX were associated with poor prognosis, whereas TMEM27 and HRH2 were linked to better outcomes. Fig. 4D illustrated the patient distribution across GMRClusters, GeneClusters, and GMRGroups. Particularly, patients in GMRCluster B and GeneCluster B exhibited elevated GMRScore (Fig. 4E, F and Table S4). In the previous section we showed that patients in GMRCluster B and GeneCluster B had a poorer prognosis, which corresponded here to the fact that patients with high GMRScores experienced poorer overall survival (Fig. 4G). The receiver operating characteristics (ROC) curve suggested that GMRScore had excellent sensitivity and specificity in predicting the survival in ccRCC patients from the four cohorts (Fig. 4H). The heatmap provided a visual representation of the contrasting gene expression patterns and clinical traits between the two GMRGroups (Fig. 4I).

### Investigation of the relationship between GMRScore and clinical features

We analyzed the relationship between GMRScore and various groups divided by different clinical features. We observed that individuals in the low GMRGroup generally had lower TNM stages and tumor grades (Fig. S3A–F). Importantly, Patients in GMRScore group were strongly associated with worse prognosis in all subgroups (Fig. S3K–P). In conclusion, the results emphasized the predictive signature’s resilience in anticipating the survival outcomes of ccRCC patients (Fig. S3).

### Establishment and validation of a nomogram

**Fig. 5:**
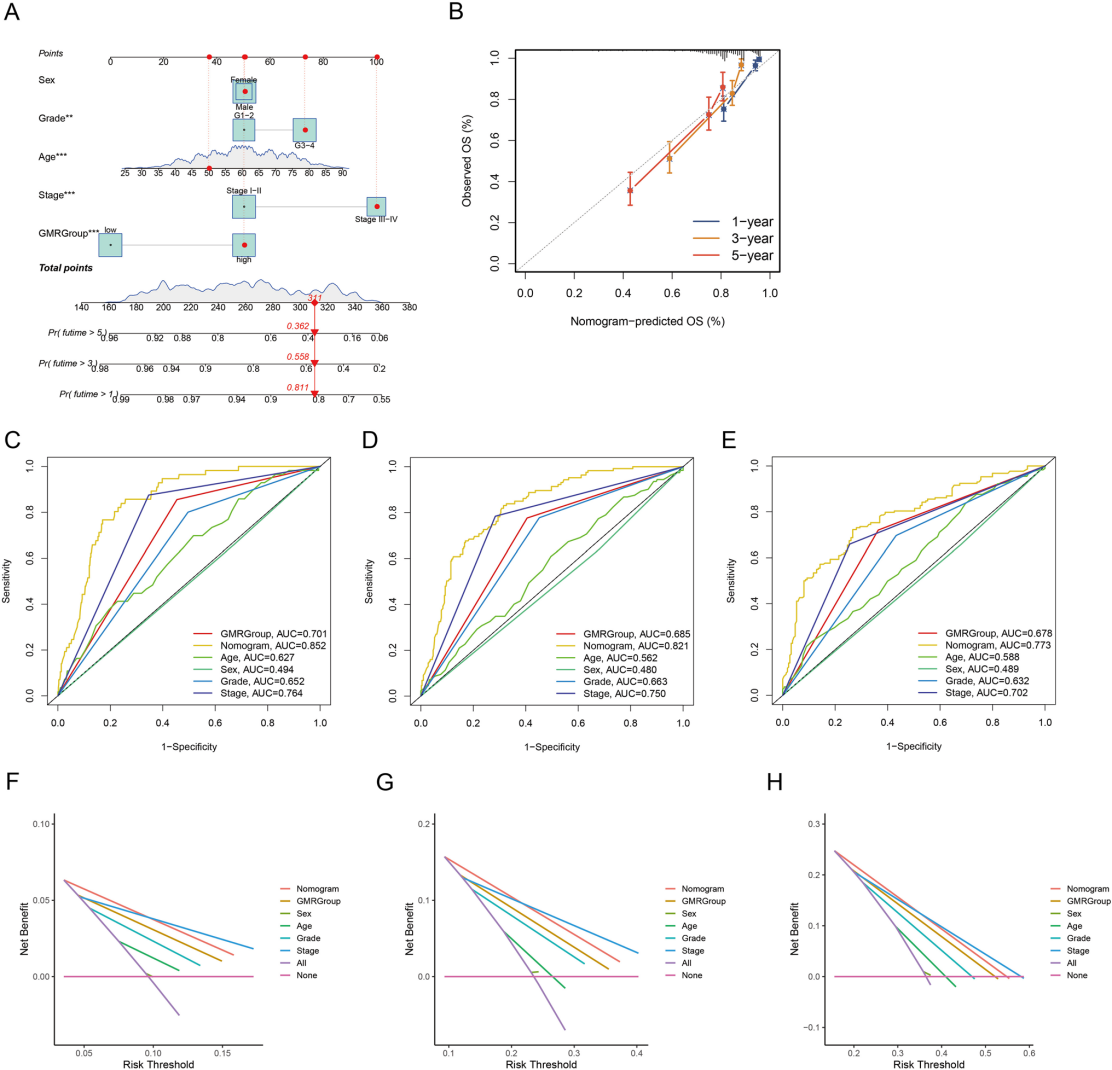
Establishment and validation of a nomogram. **A** The nomogram for predicting the 1-, 3-, and 5-year OS based on the GMRGroups and clinicopathological features. **B** Calibration curves for 1-, 3-, and 5-year survival. **C**–**E** ROC curves of the nomogram in predicting the 1-, 3-, and 5-year OS. **F–H** The decision curve analysis (DCA) of the nomogram

We integrated four prognostic factors with the GMRScore to develop a nomogram to predict OS for different lengths of time (Fig. 5A). The calibration curves for predicting OS of one, three, and five years aligned well with the actual outcomes (Fig. 5B). The ROC analysis demonstrated that risk scores significantly outperform clinicopathological features in predicting outcomes, indicating the nomogram’s outstanding prognostic performance (Fig. 5C–E). Furthermore, we observed that the nomogram provided greater net benefits for prognostic prediction (Fig. 5F–H). The results indicate that the nomogram effectively predicts ccRCC patient prognosis and could enhance precision therapy.

### Immune landscape and immunotherapy response of the GMRGroups

**Fig. 6:**
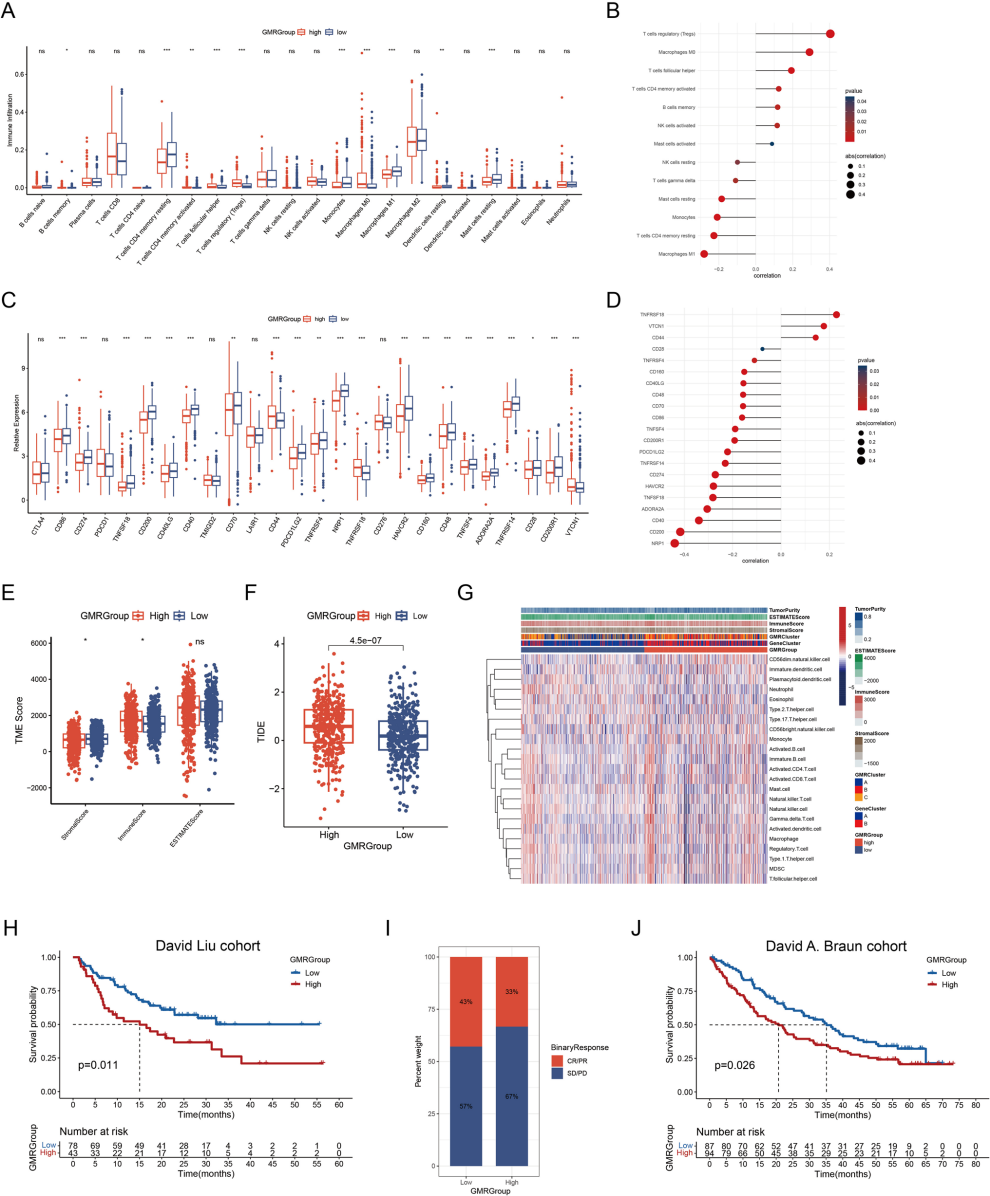
Immune landscape and immunotherapy response of the GMRScore. **A** The difference in infiltrating abundance of 22 immune cells between two GMRGroups. **B** The correlation between GMRScore and infiltrating abundance of immune cells. **C** Expression of 26 immune checkpoints (ICPs) in the high and low GMRGroups. **D** The correlation between GMRScore and expression of ICPs. **E** Correlations between GMRScore and both immune and stromal scores. **F** The differences in tumor immune dysfunction and exclusion (TIDE) score between two GMRGroups. **G** The distributions of TME score, GMRClusters, GeneClusters, GMRGroups, and the abundance of immune cell types between the two GMRGroups. **H** Kaplan–Meier curves for patients in different GMRGroups in the David Liu cohort. **I** The differences in immunotherapy response between two GMRGroups in David Liu cohort. **J** Kaplan–Meier curves for patients in different GMRGroups in the David A. Braun cohort. ns, not significant, *p < 0.05, **p < 0.01, ***p < 0.001

Heterogeneity of tumor microenvironment (TME) in different subgroups in ccRCC significantly affects the efficacy of immunotherapy, so we conducted further studies. We observed heightened infiltration of Tregs, macrophages M0, and T cells follicular helper in the high GMRGroup, exhibiting a positive correlation with the GMRScore. Conversely, macrophages M1, T cells CD4 memory resting, and monocytes were reduced within the high GMRGroup, negatively correlated with the GMRScore (Fig. 6A, B). Given the significance of immune checkpoint blockade (ICB) in clinical ccRCC treatment, we scrutinized the ICPs expression of two GMRGroups. TNFRSF18, VTCN1, and CD44 displayed elevated expression in the high GMRGroup, positively correlated with the GMRScore, while NRP1, CD200, CD40, and ADORA2A showed negatively correlated with the GMRScore (Fig. 6C, D). The ESTIMATE analysis revealed differences in levels of immunization and stromal cell infiltration in GMRGroups (Fig. 6E).

Additionally, patients in the low GMRGroup were tend to have lower TIDE scores, suggesting their increased likelihood of benefiting from ICB (Fig. 6F). Furthermore, tumor purity and ESTIMATE analysis results were presented in a heatmap (Fig. 6G). We conducted an additional evaluation to determine the predictive value of the GMRScore for ICB response using two external cohort, the David Liu cohort and David A. Braun cohort. Notably, high GMRGroup’patients demonstrated poorer prognosis, aligning with their worse response to ICB (Fig. 6H–J). In summary, the GMRScore demonstrated strong predictive capability for response to ICB in patients with ccRCC.

### ALDH18A1 exerts protumoral effects in ccRCC

**Fig. 7:**
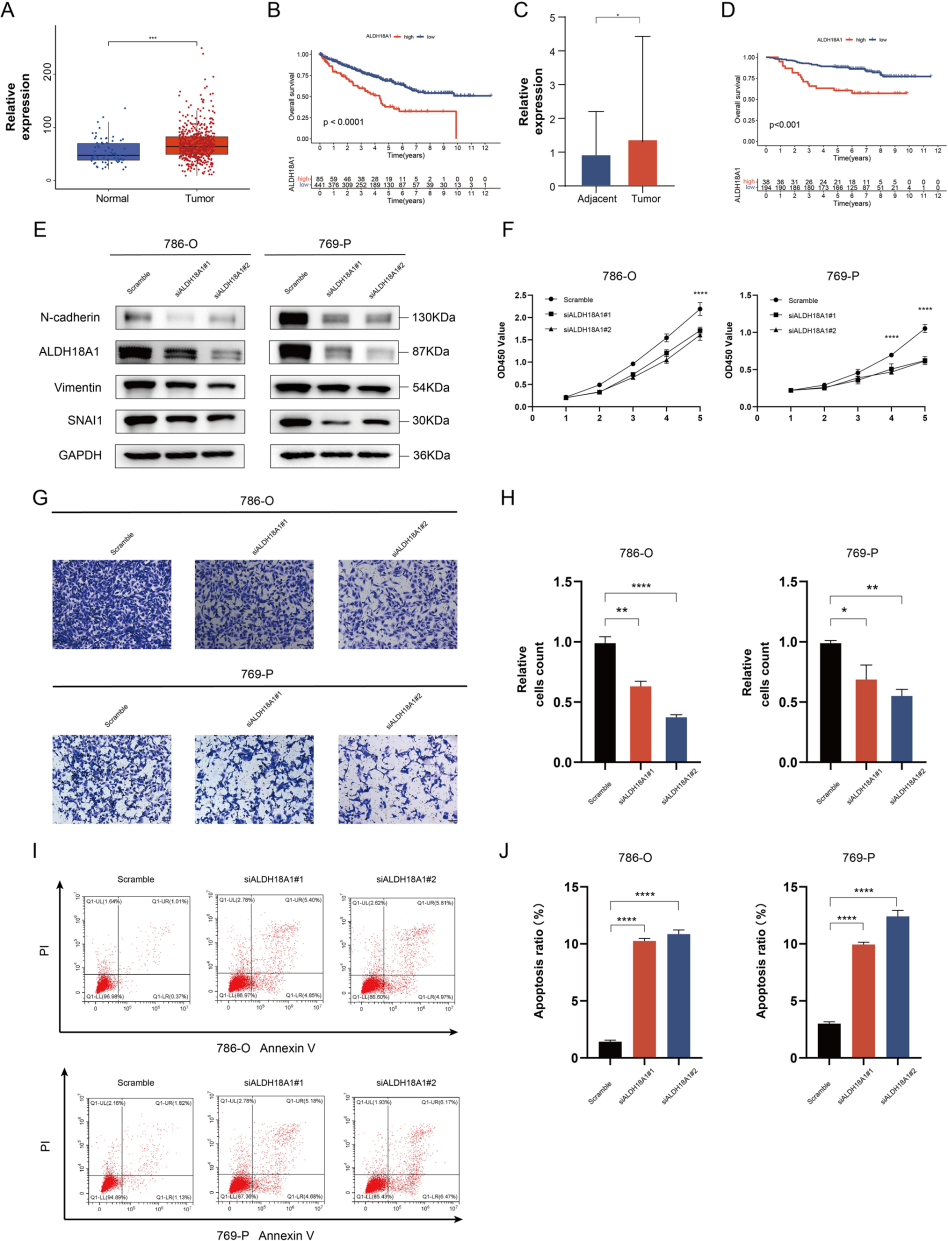
ALDH18A1 exerts protumoral effects in ccRCC. **A** Expressions of ALDH18A1 between tumor and normal specimens in the TCGA-KIRC cohort. **B** Kaplan–Meier curves for patients from the TCGA-KIRC cohort. **C** Comparative protein levels of ALDH18A1 in tumor and adjacent tissues samples from FUSCC proteomic cohort. **D** Kaplan–Meier curves for patients in two ALDH18A1 protein expression levels. **E** Western blotting for ALDH18A1, N-cadherin, SNAI1, and Vimentin protein expression level in 786-O and 769-P cells. **F** CCK-8 assays of the viability of 786-O or 769-P cells without or with ALDH18A1 knockdown at the indicated times. **G**, **H** Representative images and the statistical analysis of the results from the invasion assays with the 786-O and 769-P cells from different groups are presented (scale bar = 200 µm). **I**–**J** Percentage of apoptotic cells in 786-O or 769-P cells with or without ALDH18A1 knockdown detected by flow cytometry. ns—not significant, *p < 0.05, **p < 0.01, ***p < 0.001

ALDH18A1, a member of the GMRGs, encodes the mitochondrial enzyme P5CS, which is responsible for initiating proline biosynthesis [43]. Analysis of TCGA-KIRC cohort revealed that ALDH18A1 (P5CS) displayed elevated expression in tumor (Fig. 7A), and it was linked to shorter overall survival (Fig. 7B). Analysis of the FUSCC proteomic cohort revealed that ALDH18A1 was overexpressed in tumor (Fig. 7C), and once again, patients with elevated expression experienced a shorter OS (Fig. 7D). So, we hypothesized that it plays a protumoral role in ccRCC and conducted further exploration.

Through in vitro experiments, we delved into the relationship between ALDH18A1 and the ccRCC malignant phenotype. 786-O and 769-P were transfected with ALDH18A1 small interfering RNA (siRNA) to silence its expression, and the knockdown efficiency was validated through western blotting (Fig. 7E). Furthermore, we examined the expression of several most well-known EMT-related genes (N-cadherin, SNAI1, Vimentin) and observed that ALDH18A1 knockdown suppressed the EMT process, indicating a reduced capacity for tumor migration (Fig. 7E). The CCK-8 indicated knockdown of ALDH18A1 notably inhibited tumor cell proliferation (Fig. 7F). Transwell invasion assay revealed that knockdown of ALDH18A1 inhibited tumor cell invasion (Fig. 7G, H). Additionally, ALDH18A1 knockdown enhanced cell apoptosis (Fig. 7I, J).

## Discussion

Metabolic deregulation serves as a significant factor in progression of numerous cancers [44]. ccRCC has been extensively acknowledged as a complex disease with its development being influenced by numerous factors. In the investigation of the mechanism of its progression, metabolic reprogramming should be considered as a key factor, because it affects many life activities of tumor cells and has a huge impact on the heterogeneity of tumor cells and TME [45]. Therefore, metabolic reprogramming of ccRCC should receive greater attention, given that the disease’s heterogeneity poses challenges to clinical management. Notably, ccRCC is characterized by disturbances in glucose metabolism and oxidative phosphorylation, along with distinctive metabolic adaptations, including heightened reliance on glutamine [46]. Research indicates that ccRCC exhibits a higher rate of glutamine consumption compared to normal renal tissue [46, 47]. Studies have demonstrated that certain human tumors and cancer cell lines [48–50], such as some ccRCC cells [51, 52], exhibit a reliance on exogenous glutamine. When glutaminase is inhibited, these cells show increased mortality in vitro, indicating glutamine addiction. Our study indicates that multiple enzymes and biomarkers associated with glutamine metabolism are disrupted in ccRCC. Hence, investigating the effective classification of genes involved in glutamine metabolism is likely to be highly beneficial for the prognosis and clinical treatment of ccRCC.

In our research, we utilized the expressions of 74 glutamine metabolism related genes (GRMGs) to stratify ccRCC patients into three distinct glutamine metabolism patterns (GMRCluster A, B, and C) using the consensus clustering algorithm. The three clusters exhibit notable differences in prognosis, metabolic pathways, and tumor microenvironment (TME). GMRCluster A, characterized by the most favorable prognosis, was marked by elevated expression of GMRGs, activation of amino acid metabolism pathways, a higher degree of tumor purity, a lower Tregs infiltration, and a higher infiltration of M1 macrophages. To further explore the underlying molecular mechanisms of different glutamine metabolism patterns, we identified prognosis-related differentially expressed genes among the three GMRClusters. Clustering of these genes that overlaped between major gene clusters produced two GeneClusters that facilitated the identification of glutamine metabolism related gene classification. Similar to the clustering patterns observed in GMRCluster, these genetic clusters also displayed clear differences. GeneCluster A, which had a better prognosis, was marked by upregulated DEGs expression, amino acid pathway activation, and enhanced tumor purity. In addition, the low infiltration levels of Tregs and neutrophils, coupled with high M1 macrophage levels, indicated more active anti-tumor immunity in TME. The findings suggest that varying glutamine metabolism patterns contribute to the establishment of intricate TMEs, which in turn influence the survival outcomes of ccRCC patients. Furthermore, we used six genes to construct a GMRscore to divide patients into two groups to predict prognosis and response to ICB. Patients with low GMRScore had longer OS and benefited more from ICB, which was validated by external ICB-treated cohorts including 181 accRCC patients treated with nivolumab (anti-PD-1). The prognostic value of glutamine metabolism prognostic signature has been investigated in various cancers [53–55], yet remains not widely understood in ccRCC. Our study aims to contribute to this area of research. Despite both studies examining the connection between glutamine metabolism genes and ccRCC, our research employed distinct scoring model [56]. Briefly, we created three GMRClusters by clustering GMRGs and analyzed their DEGs to establish a GMRScore. Patients with high GMRScore exhibited a worse prognosis. In addition, GMRscore may facilitate the selection of patients who are more likely to respond to immunotherapy and offer insights into survival prognostication, with potential clinical applications.

Glutamine is typically considered a nonessential amino acid, providing carbon and nitrogen for tumor cells. However, under certain physiological and pathological conditions, such as during immune responses and in tumors, it can become conditionally essential amino acid [57]. Glutamine’ metabolite glutamate can be used as one of the raw materials for the synthesis of glutathione, and together with NADPH, it can be used as a reducing agent to maintain intracellular redox homeostasis, which is conducive to normal cell proliferation. GLS, the initial and rate-limiting enzyme in glutamine catabolism, has two isoforms in mammals: kidney-type glutaminase (GLS1) and liver-type glutaminase (GLS2) [58]. In various cancers, GLS1 overexpression is frequently associated with tumor aggressiveness, malignancy, and prognosis [59–61]. Our research confirmed that elevated GLS1 expression in renal cancer correlates with poor patient outcomes. Moreover, in a phase II clinical trial, Telaglenastat (CB-839), a selective GLS1 inhibitor, in combination with Everolimus, demonstrated promising therapeutic effects in patients with RCC [62].

ALDH18A1 encodes the mitochondrial enzyme P5CS, which is responsible for initiating proline biosynthesis [43]. P5CS catalyzes the conversion of glutamate to pyrroline-5-carboxylate (P5C), an essential intermediate in the proline biosynthesis pathway. Downregulation of P5CS leads to a complete cessation of proline production and secretion [43]. P5CS utilizes glutamate, a key product of glutamine catabolism, in its enzymatic reaction, thereby playing a pivotal role in the regulation of glutamine metabolism.

Research has elucidated the pivotal contribution of proline metabolism to tumor progression. Proline, a non-essential amino acid, modulates intracellular ROS levels through its metabolic rate, influencing cytokine secretion and discharge by tumor cells, which contributes to immune evasion in non-small cell lung cancer [63]. The proline metabolic pathway intermediate P5C has been shown to suppress T cell proliferation and activation [64]. Therefore, proline metabolism is identified as a significant factor in tumor development. Pyrroline-5-carboxylic acid reductase-1 (PYCR1), a pivotal enzyme in the glutamate-to-proline biosynthetic pathway, is markedly upregulated across numerous tumors [65]. It enhances proliferation, invasion, and migration of carcinoma by modulating various signaling pathways, thereby contributing to an unfavorable prognosis. Elevated PYCR1 expression is correlated with exhausted T cells and indicates an unfavorable OS for patients with ccRCC [66], which was validated in our cohorts. In contrast, the role of P5CS, the rate-limiting enzyme in the glutamate-to-proline biosynthetic pathway, remains unexplored in ccRCC. The transcriptomic analysis of four databases indicated that ALDH18A1 exhibited elevated expression in tumor cells and was linked to a reduced overall survival. A cohort of 232 ccRCC cases from FUSCC demonstrated its upregulation at the protein level and its close association with poor prognosis. In the ALDH18A1 knockdown group, the protein expression of two well-known EMT-related genes (N-cadherin and Vimentin) and the Epithelial mesenchymal transition (EMT) transcription factor Snail1 decreased, suggesting its suppression of EMT and reduction of tumor cell migration properties [67]. Furthermore, in the ALDH18A1 knockdown group, the proliferation and invasion capabilities of human ccRCC cells were inhibited, while apoptosis increased. Hence, ALDH18A1 exerts a protumoral role in ccRCC. We suggest that ALDH18A1 serves as a prognostic biomarker and a promising therapeutic target for ccRCC.

It is important to recognize several limitations inherent in our research. Firstly, the results were retrospectively derived from publicly available data, which inherently carries the risk of selection bias and confounding factors. To strengthen the validity and universality of these results, they should be validated using a prospective study design. Additionally, the foundation for reliability of GMRscore to predict the immunotherapy efficacy may not be particularly robust due to the lack of sufficient validation in large-scale immunotherapy cohorts with transcriptomic data in ccRCC. Thirdly, while the study has identified a potential role for ALDH18A1 in promoting malignant phenotype of tumors, the behind precise biological mechanisms remain to be fully elucidated, which is a possible direction for future research.

## Conclusion

In summary, this research systematically deciphered the glutamine metabolism patterns of ccRCC. The GMRScore emerged as an ideal predictor of patients’ prognosis and ICB response. In addition, this work explored for the first time the protumoral role of ALDH18A1, a potential therapeutic target, in ccRCC.

## Supplementary Information

Below is the link to the electronic supplementary material.

Supplementary file1 (JPG 1899 KB)

Supplementary file2 (JPG 1245 KB)

Supplementary file3 (JPG 1699 KB)

Supplementary file4 (XLSX 638 KB)

## Data Availability

The original data are provided within the article and its supplementary materials. For any additional inquiries, please contact the corresponding authors. The authors are prepared to share the raw data that underpins the conclusions of this study, subject to reasonable conditions.

## Abbreviations

accRCC: Advanced clear cell renal cell carcinoma
ccRCC: Clear cell renal cell carcinoma
CDF: Cumulative distribution function
CNVs: Copy number variations
CPTAC: Clinical proteomic tumor analysis consortium
DEGs: Differential expression genes
EMBL: European molecular biology laboratory
EMT: Epithelial mesenchymal transition
FDR: False discovery rate
FUSCC: Fudan University Shanghai Cancer Center
GO: Gene ontology
GMRGs: Glutamine metabolism related genes
GSVA: Gene set variation analysis
ICGC: International Cancer Genome Consortium
ICIs: Immune checkpoint inhibitors
ICB: Immune checkpoint blockade
ICPs: Immune checkpoints
KEGG: Kyoto encyclopedia of genes and genomes
LASSO: Least absolute shrinkage and selector operator
ORR: Overall response rate
OS: Overall survival
PCA: Principal component analysis
PPI: Protein protein interaction
PFS: Progression-free survival
RCC: Renal cell carcinoma
ROC: Receiver operating characteristic
ROS: Reactive oxygen species
ssGSEA: Single-sample gene set enrichment analysis
TCGA: The cancer genome atlas
TIDE: Tumor immune dysfunction and exclusion
TME: Tumor microenvironment
