# Supplementary figures and images for: Deciphering glutamine metabolism patterns for malignancy and tumor microenvironment in clear cell renal cell carcinoma
Source: Clin Exp Med. 2024 Jul 6;24(1):152. doi: 10.1007/s10238-024-01390-4 (PMC11227463; doi:10.1007/s10238-024-01390-4)

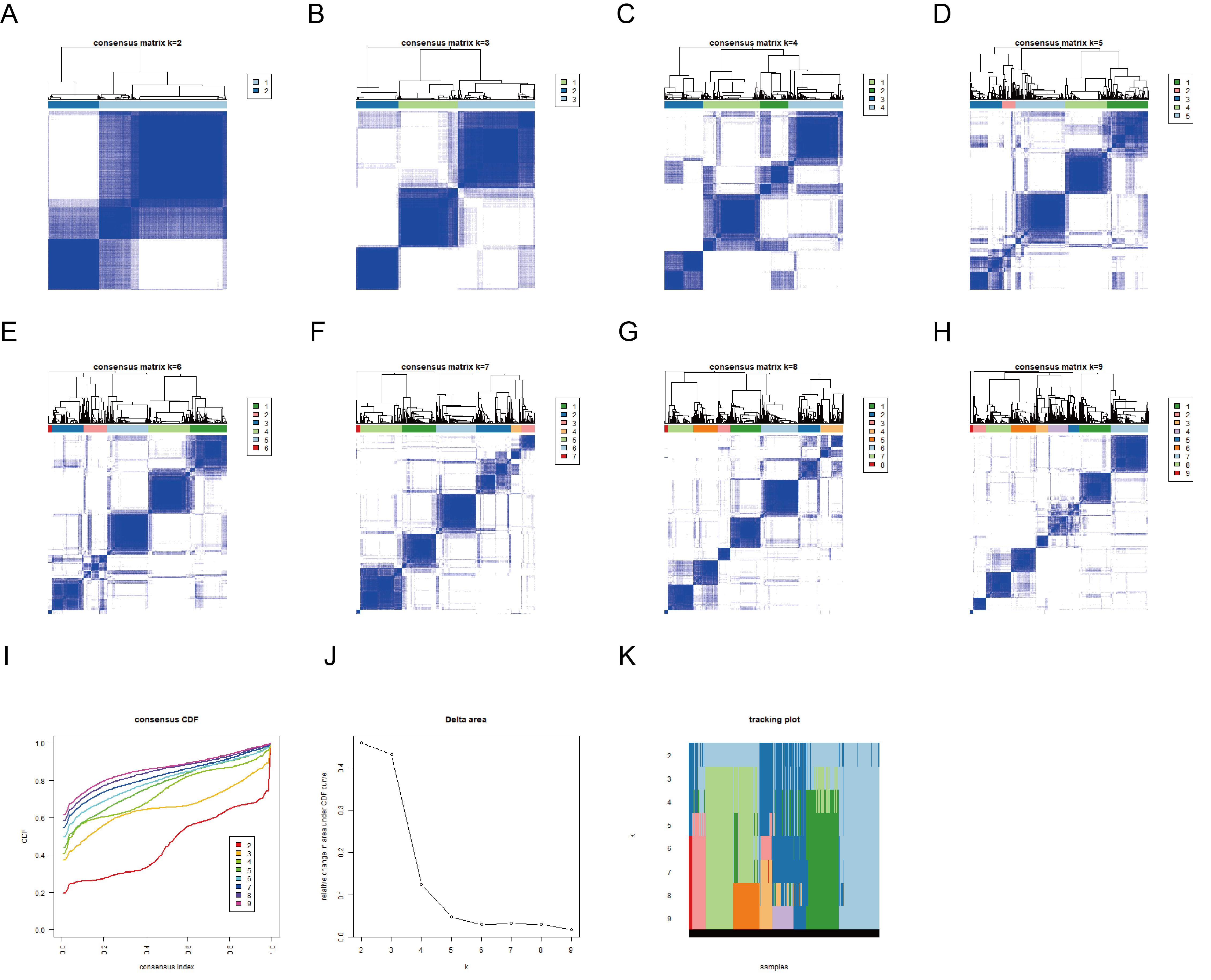

Supplement: Supplementary file 1 — Supplementary file1 (JPG 1899 KB) [file 10238_2024_1390_MOESM1_ESM.jpg]

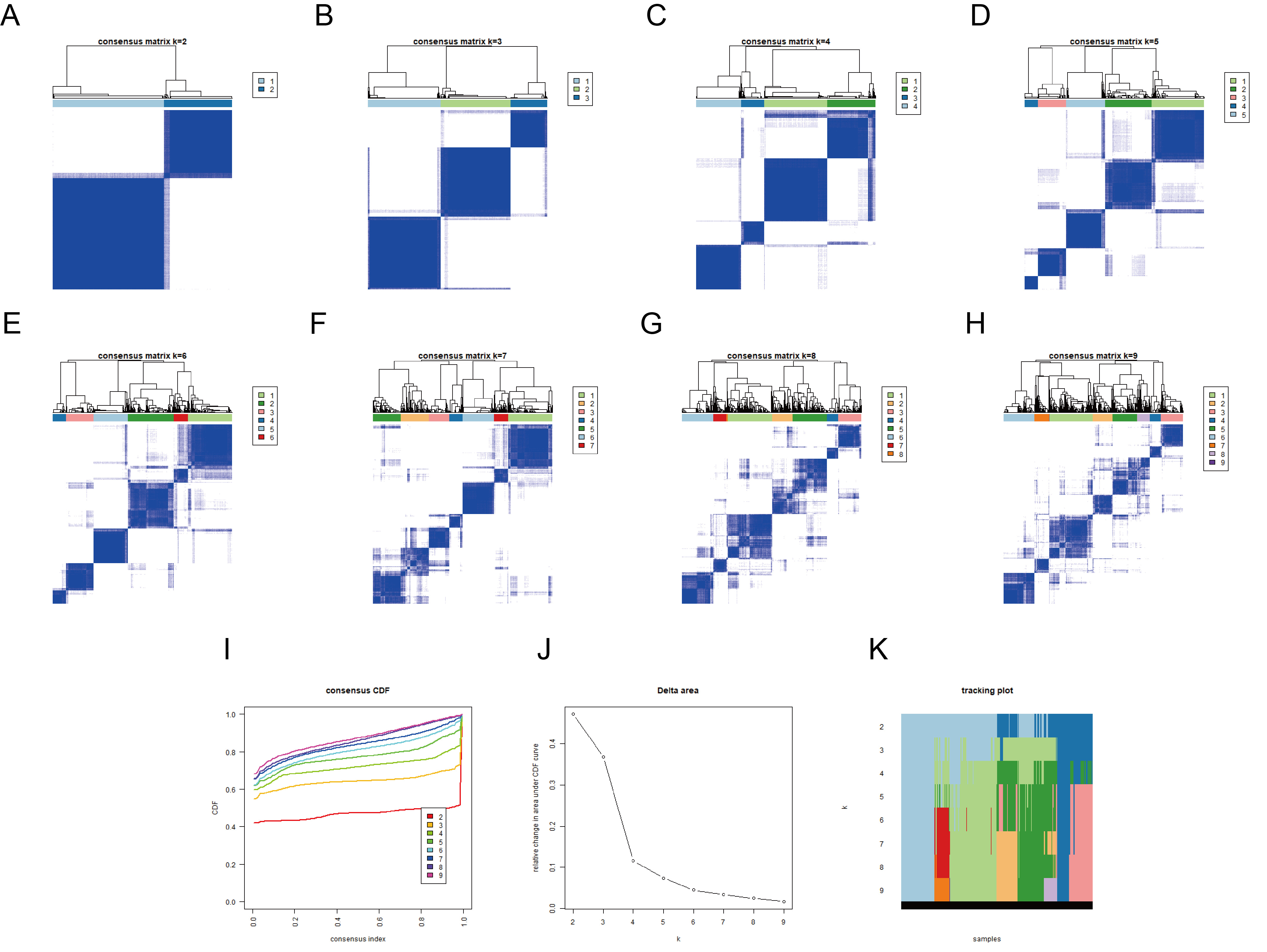

Supplement: Supplementary file 2 — Supplementary file2 (JPG 1245 KB) [file 10238_2024_1390_MOESM2_ESM.jpg]

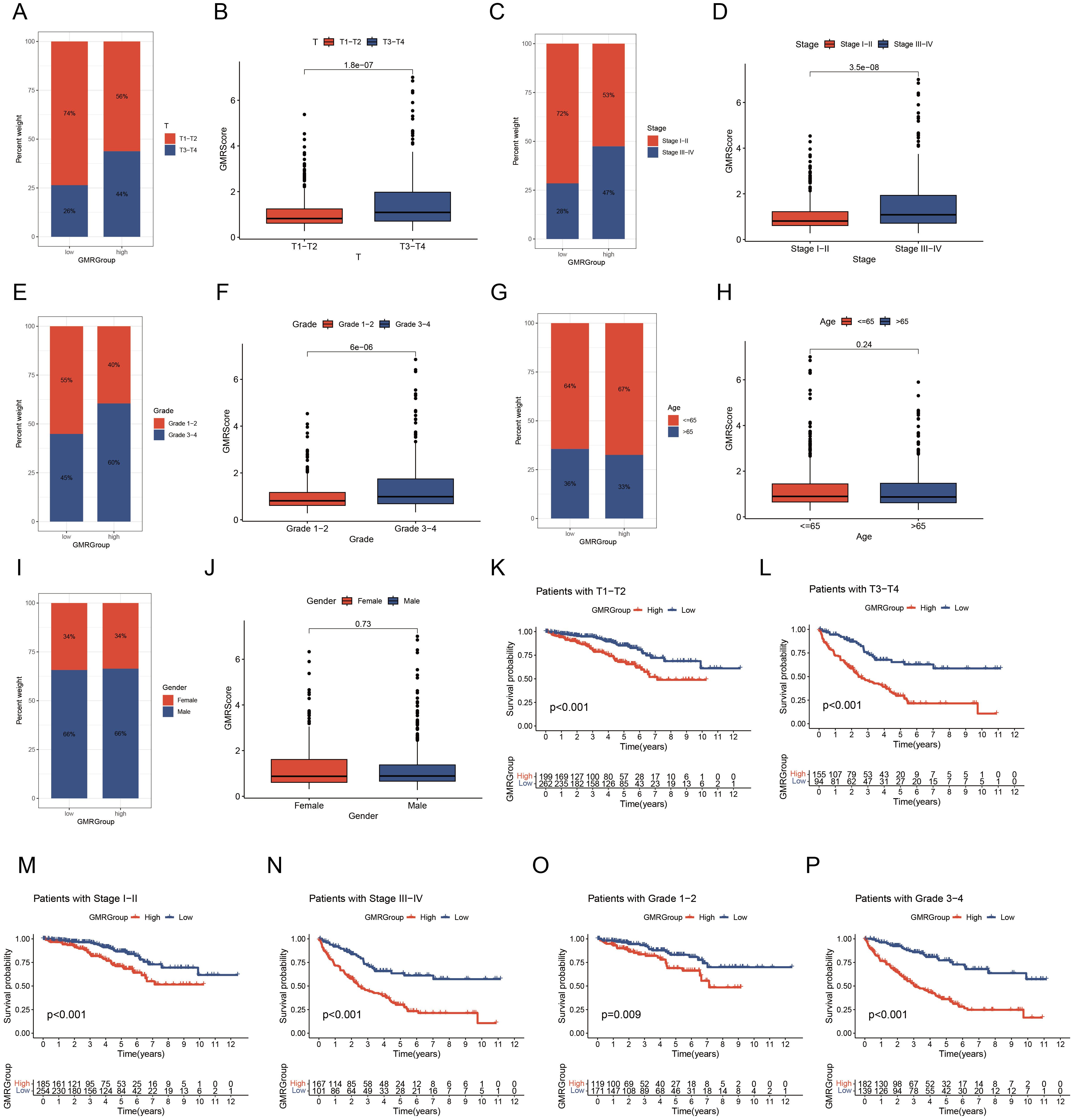

Supplement: Supplementary file 3 — Supplementary file3 (JPG 1699 KB) [file 10238_2024_1390_MOESM3_ESM.jpg]
